# Liposomal Conjugates for Drug Delivery to the Central Nervous System

**DOI:** 10.3390/pharmaceutics7020027

**Published:** 2015-04-01

**Authors:** Frieder Helm, Gert Fricker

**Affiliations:** Department of Pharmaceutical Technology and Biopharmacy, Institute of Pharmacy and Molecular Biotechnology, University of Heidelberg, Im Neuenheimer Feld 329, 69120 Heidelberg, Germany; E-Mail: gert.fricker@uni-hd.de

**Keywords:** liposomes, blood–brain barrier, cationized bovine serum albumin, liposomal conjugates, brain capillary endothelial

## Abstract

Treatments of central nervous system (CNS) diseases often fail due to the blood–brain barrier. Circumvention of this obstacle is crucial for any systemic treatment of such diseases to be effective. One approach to transfer drugs into the brain is the use of colloidal carrier systems—amongst others, liposomes. A prerequisite for successful drug delivery by colloidal carriers to the brain is the modification of their surface, making them invisible to the reticuloendothelial system (RES) and to target them to specific surface epitopes at the blood–brain barrier. This study characterizes liposomes conjugated with cationized bovine serum albumin (cBSA) as transport vectors *in vitro* in porcine brain capillary endothelial cells (PBCEC) and *in vivo* in rats using fluorescently labelled liposomes. Experiments with PBCEC showed that sterically stabilized (PEGylated) liposomes without protein as well as liposomes conjugated to native bovine serum albumin (BSA) were not taken up. In contrast, cBSA-liposomes were taken up and appeared to be concentrated in intracellular vesicles. Uptake occurred in a concentration and time dependent manner. Free BSA and free cBSA inhibited uptake. After intravenous application of cBSA-liposomes, confocal fluorescence microscopy of brain cryosections from male Wistar rats showed fluorescence associated with liposomes in brain capillary surrounding tissue after 3, 6 and 24 h, for liposomes with a diameter between 120 and 150 nm, suggesting successful brain delivery of cationized-albumin coupled liposomes.

## 1. Introduction

Access for drugs to the central nervous system (CNS) is highly restricted due to the presence of the blood–brain barrier (BBB). Consisting mainly of the capillary endothelial cells connected via *tight junctions*, it prevents the exchange of compounds between CNS and blood. Essential nutrients for CNS function are transported by membrane carrier proteins, such as the glucose transporter or amino acid carrier proteins. Thus, homeostasis of the cerebral interstitial fluid is guaranteed [1,2].

The exceptional barrier function of the BBB, apart from the *tight junctions*, is provided by ABC export proteins in the luminal membrane of the capillary endothelial cells, e.g., P-glycoprotein (P-gp, ABCB1), breast cancer resistance protein (BCRP, ABCG2) or the multi-drug resistance protein family (MRPs). Though lipophilic compounds can pass membranes through passive diffusion, these transporters recognize most of them as xenobiotica and convey them back into the blood. Many agents, e.g., morphine [3] and phenytoin [4], are substrates for P-gp which reduces their availability in the CNS drastically. For many CNS related diseases, this constitutes a major problem.

Utilizing the existing carrier proteins for nutrient delivery across the BBB is a promising option to achieve or increase availability in the brain. However, a direct coupling of carrier substrates and drugs may change the molecular makeup of both, which may reduce their uptake. Further, only small drug-to-vector ratios are possible and the use of colloidal carriers is more promising. Encapsulation of drugs into vesicles, which carry the vector on their surface leads to high drug-to-vector ratios [5,6] and the drug is protected inside the vesicle [7]. On the downside, release from inside the vesicles can be an issue and the chosen transport pathway has to be capable of endocytosis and transcytosis across the BBB.

Liposomes possess several advantages. Their composition is very flexible and can easily be adapted to suit their purpose. Their constituents are biocompatible and biodegradable [8]. The introduction of sterically stabilized (PEGylated) lipids can increase plasma half-life and prevent phagocytosis through the mononuclear phagocyte system [9,10]. Under various temperatures and lower pH values, unstable lipids or lipid compositions incorporated into the liposomal membrane can also facilitate release under these conditions [11,12,13,14].

As possible vectors to be coupled to the surface of liposomes, transferrin and apolipoprotein E4 (apoE4) are of interest, which are being transported into the brain via the transferrin- and low-density lipoprotein receptors, respectively. Both receptors are highly expressed at the BBB [5,15,16]. Previous studies showed that liposomes conjugated with an antibody against the transferrin receptor are able to cross the BBB [5]. Liposomes, coupled with the signal sequence bearing the apoE4 peptide, were also shown to be taken up by brain capillary endothelial cells (BCEC) *in vitro* [17]. Aside from receptor mediated endocytosis, adsorptive mechanisms are possible. Transcytosis of cationized serum albumin through BCEC is known to occur *ex vivo* and *in vivo* [18,19] and we showed uptake of liposomes conjugated with cationized bovine serum albumin (cBSA) into BCEC *in vitro* and *ex vivo* [6]. A closer look at the uptake of these cBSA-liposomes *in vitro* and *in vivo* will be presented here.

## 2. Experimental Section

### 2.1. Materials

Phospatidylcholine from egg yolk (EPC) and distearylphosphatidylethanolamine-methoxypolyethylene glycol-2000 (DSPE-MPEG) were obtained from Lipoid GmbH (Ludwigshafen, Germany). Distearylphosphatidylethanolamine-polyethylene glycol-2000 maleimide (DSPE-PEG-MI) and dipalmitoylphosphatidylethanolamine-lissamine rhodamine B (DPPE-RH) were purchased from Avanti Polar Lipids, Inc. (Alabaster, AL, USA). Cholesterol, bovine serum albumin (BSA), ethylenediamine dihydrochloride, 1-ethyl-3-(dimethylaminopropyl)-carbodiimide hydrochloride (EDAC) were from Sigma–Aldrich (St. Louis, MO, USA) and *N*-succinimidyl-*S*-acetyl-thioacetate (SATA) was from AppliChem GmbH (Gatersleben, Germany). All non listed chemicals were purchased from commercial suppliers at analytical grade.

### 2.2. Liposome Preparation

Lipid solutions in chloroform/methanol 9:1 were mixed with EPC 54 mol%, cholesterol 40 mol%, DSPE-MPEG 5 mol% and DSPE-PEG-MI 1 mol%. For fluorescence detection, additionally, DPPE-RH was added to 0.1 mol% for *in vitro* and 0.6 mol% for *in vivo* experiments. Solvents were evaporated under nitrogen flow at 50 °C and the lipid film dried in vacuum for at least 30 min [20]. The mass of the dried lipid film was determined and an equal amount of glass beads (ø 0.75–1 mm) and the 1.5fold mass in phosphate buffered saline (PBS) were added. Then, the mixture was subjected to dual asymmetric centrifugation (DAC) [21] (30 min, 3540 rpm). Subsequently, the 2.5fold mass in PBS of the original lipid film was added to the vesicular phospholipid gel and subjected to another DAC run (1 min, 3540 rpm). The last two steps were repeated and the resulting liposomal dispersion was diluted to a lipid concentration of 100 mM. The hydrodynamic radius and zeta-potential of the vesicles were determined using dynamic light scattering (DLS) in a Zetasizer Nano ZS^®^ (Malvern Instruments, Worcs, UK). Actual lipid concentrations were measured with HPLC (UltiMate 3000; Acclaim^®^ RP-18 column, Dionex, Dreieich, Germany).

### 2.3. Synthesis of Sulfhydrated Cationized BSA (cBSA-SH)

cBSA was prepared after a modified version of the method of Hoare and Koshland [22]. To a solution of ethylenediamine (3 M, pH 4.75), a solution of bovine serum albumin (BSA, 5 mM) in PBS and EDAC (30fold molar excess regarding BSA) was added. The reaction mixture was stirred at room temperature for 2 h and then stopped through addition of sodium acetate buffer (4 M, pH 4.75). Separation of cBSA and surplus ethylenediamine, as well as washing of cBSA with PBS, was achieved with filtration through membranes with 30 kDA molecular weight cut off. Concentration of the protein solution was determined through UV-spectrometry against a BSA calibration curve. Sulfhydration was achieved using the agent *N*-succinimidyl-*S*-acetylthioacetat (SATA) [23] in fivefold excess regarding cBSA and incubation over night in sulfhydration buffer (HEPES 10 mM, EDTA 2 mM, NaCl 150 mM, pH 7.4). Purification of cBSA-SH was again carried out by membrane filtration. Final protein concentrations were determined via BCA assay. Quality and integrity of the modified protein were checked with SDS-PAGE and the extent of the cationization through isoelectric focussing. Determination of free sulfhydryl groups was performed via Ellman’s test [24]. Until use, the protein solution was freeze dried and stored at −20 °C.

### 2.4. Covalent Coupling of Liposomes with cBSA

Freeze dried cBSA was reconstituted in PBS to a concentration of 5 mM and the protective group of the sulfhydryl group removed by hydroxylamine (100 mM). Afterwards, it was combined with freshly prepared liposomes containing the linker lipid DSPE-PEG-MI. The mixture was incubated over night. Separation of liposomes and uncoupled protein was achieved through size exclusion chromatography (Sepharose^®^ CL-4B). Since regular protein assays fail to perform in the presence of lipids [25,26], and the turbidity of liposomal dispersions disturbs photometric measurements, the coupling efficiency, *i.e.*, the percentage of occupied linker lipids, was assessed with fluorescein labelled cBSA (FITC-cBSA). Fluorescent measurements were performed with a plate reader (Fluoroscan Ascent, Thermo Fisher). Vesicle diameter and zeta potential of the liposomal dispersions were measured before and after the coupling procedure.

### 2.5. Isolation and Cultivation of Brain Capillary Endothelial Cells

Brain capillary endothelial cells from pigs (PBCEC) were prepared as previously described [27] with the following modifications. As a serum additive to culture medium, newborn calf serum was used. Flaming of brain hemispheres was omitted. To destroy any remaining pericytes, puromycin (0.05% *v*/*v*) was added for the first day of culture. Dead cells were washed out with PBS containing Ca^2+^ and Mg^2+^ (0.9 and 0.5 mM, respectively) on the day after isolation. On the second day, PBCEC were detached by trypsin solution (0.0625% in PBS) and frozen in serum with 10% DMSO at −80 °C. Five days before the experiments, PBCEC were thawed and seeded at 250,000 cells per cm^2^. Two days after seeding, the cells were switched to serum free medium.

### 2.6. In Vitro Uptake of cBSA-Liposomes

Uptake of liposomes into PBCEC was examined via an assay previously established [6]. In brief, PBCEC were incubated with fluorescent liposomal conjugates in uptake buffer (HEPES 15 mM, NaCl 103 mM, KCl 4.7 mM, CaCl_2_ 2.5 mM, KH_2_PO_4_ 1.2 mM, MgSO_4_ 1.2 mM, glucose 10 mM, pH 7.4) in different concentrations over various time periods. After incubation, PBCEC were washed with 4 °C cold uptake buffer and submitted to washing with acidic buffer (citric acid 26 mM, Na_3_citrate 9.2 mM, NaCl 90.1 mM, KCl 30 mM, pH 3) to remove the remaining surface bound liposomes [28]. The cells were then lysed with an uptake buffer containing 1% (*v*/*v*) Triton X-100 over 30 min at 60 °C. The liposomal fluorescence in the cell lysate was detected with a fluorescence plate reader (Fluoroscan Ascent, Thermo Fisher, Dreieich, Germany) at wavelengths of 530 and 590 nm for excitation and emission, respectively.

### 2.7. In Vivo Uptake of cBSA-Liposomes

cBSA-liposomes were injected intravenously into the tails of male Wistar rats with a body weight between 200 and 250 g. Doses were between 11 and 25 mg lipid per kg body weight and were applied in a volume of no more than 500 µL. Three animals were used for each experiment. Fifteen minutes prior to sacrificing them, 500 µL of a FITC-BSA solution (4% *w*/*v*) was injected into the tail veins to stain the capillaries. After 1, 3, 6 and 24 h, respectively, the animals were sacrificed and the brains excised. The excised organs were washed in ice cold brine (0.9% *w*/*v*), shock frozen in isopentane with dry ice and then stored at −80 °C. All procedures performed on animals were approved by an ethics committee of the Regierungspräsidium Karlsruhe (file-number: 35-9185.81/G-38/11) and carried out in accordance with FELASA and German law.

### 2.8. Cryosections of Animal Tissue

Cryosections were prepared at −20 °C (CM3050 S, Leica Biosystems, Wetzlar, Germany). The organs were cut in 25 µm thin slices and transferred onto adhesion slides. After 15 min of drying at room temperature, the slices were fixed over night with a solution of mowiol 4-88 and then stored at −20 °C.

### 2.9. Confocal Laser Scanning Fluorescence Microscopy

In order to determine the actual uptake of liposomal conjugates into PBCEC, cells were cultivated in microscopy dishes (Cellview^®^, Greiner Bio-One, Frickenhausen, Germany) and incubated with fluorescent liposomal conjugates as described above, exempt cell lysis. Cells were then inspected with a confocal laser scanning microscope (TCS SP5 X, Leica Microsystems, Wetzlar, Germany) under live conditions (37 °C, 5% CO_2_) with wavelengths of 561 nm for excitation and 570–610 nm for emission.

The examination of cryosections was performed under non live conditions and with sequential detection of fluorescein (capillaries) and rhodamine (liposomes) at 496/500–520 and 561/570–610 nm for excitation/emission, respectively.

## 3. Results and Discussion

### 3.1. Liposome Preparation and Coupling Efficiency

Liposomes prepared by the film method with subsequent DAC had an average diameter between 118.2 ± 0.3 and 185.8 ± 1.7 nm with polydispersity indices (PDI) below 0.2 as determined by dynamic light scattering. Isoelectric focusing showed that cationization of BSA led to an increase of pI from native 4.9 to approximately 9.0 [29]. The determination of introduced sulfhydryl groups via Ellman’s test resulted in 2.8 ± 0.2.

Protein coupling increased liposome diameter by 3.7 ± 1.5 to 50.9 ± 1.1 nm. Since cBSA has a hydrodynamic radius of 3.48 nm, this increase cannot be explained by protein addition alone [29]. Oligomerisation of liposomes through proteins containing multiple sulfhydryl groups is possible, though it seems unlikely, since the increase in size is not high enough. However, an increase through influx of buffer during the coupling and subsequent purification steps may have occurred.

Coupling efficiency was examined with FITC-cBSA. The linker lipid DSPE-PEG-MI was incorporated into liposomes to molar percentages of 0.25, 0.5 and 1 in respect to the total lipid amount. FITC-cBSA was added in ratios towards linker of 1:1, 2.5:1 and 5:1. Since the incorporation of DSPE-PEG-MI during liposome formation happens randomly, and statistically half of the linker lipids are facing inwards, coupling efficiencies around 50% should be the achievable maximum. Protein:linker ratios of 1:1 and 2.5:1 delivered coupling efficiencies around 10% and 30%, respectively. A 5:1 ratio led to efficiencies between 40% and 70% which corresponds to a maximum yield of the coupling reaction. Exact values are given in Table 1.

**Table 1:** Coupling efficiency in respect to protein:linker ratio and linker concentration. Efficiency values are mean ± standard deviation (*n* = 3).

Protein:Linker Ratio	DSPE-PEG-MI Concentration	FITC-cBSA Concentration (mM)	Coupling Efficiency (%)
1:1	0.25 mM (0.25%)	0.25	10.6 ± 1.5
	0.50 mM (0.5%)	0.50	26.5 ± 9.1
	1.0 mM (1%)	1.0	72.0 ± 13.0
2.5:1	0.25 mM (0.25%)	0.625	10.7 ± 3.8
	0.50 mM (0.5%)	1.25	30.3 ± 2.9
	1.0 mM (1%)	2.5	42.0 ± 9.9
5:1	0.25 mM (0.25%)	1.25	10.8 ± 0.8
	0.50 mM (0.5%)	2.5	36.1± 0.9
	1.0 mM (1%)	5.0	63.5 ± 0.5

### 3.2. In Vitro Uptake of cBSA-Liposomes

**Figure 1:**
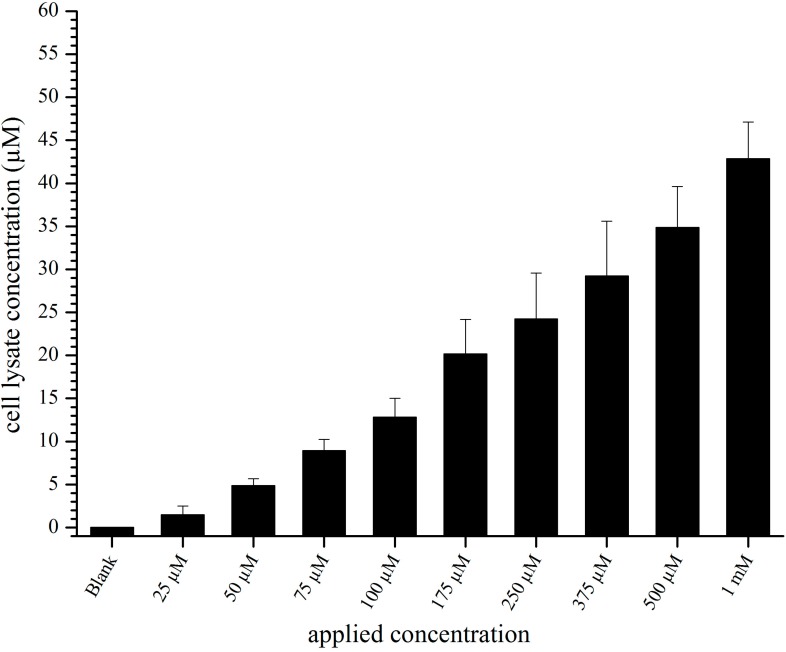
Liposomal concentration in cell lysate after incubation with cationized bovine serum albumin (cBSA)-liposomes for 2 h. Values are the means of 3 separate experiments ± standard deviation (*n* = 8, each).

The measurement of liposomal concentrations by fluorescence in cell lysates of PBCEC incubated with increasing concentrations of cBSA-liposomes is shown in Figure 1. Increasing concentrations of applied liposomes led to an increased fluorescence in the lysate. The maximum of applied liposomes (1 mM) yielded liposomal concentrations between 42.8 ± 4.4 µM.

**Figure 2:**
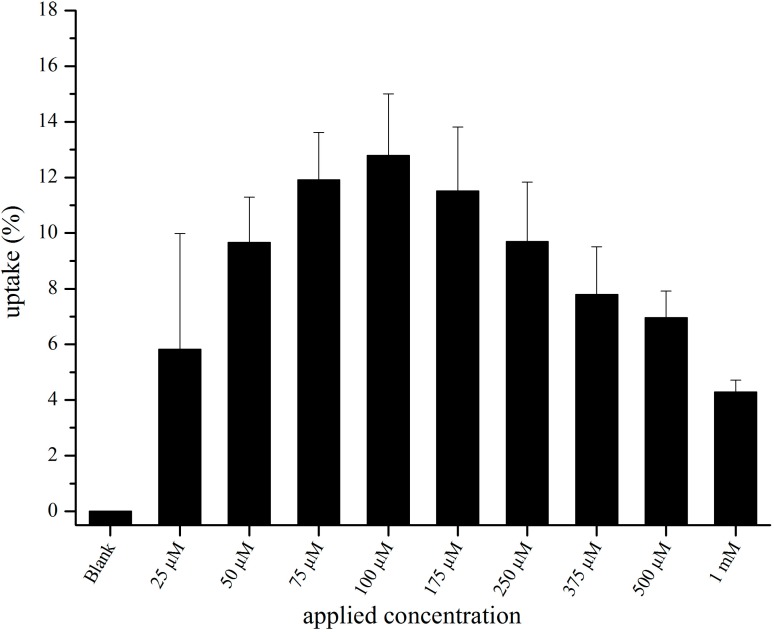
Percentage of taken up liposomes after incubation with cBSA-liposomes for 2 h. Values are the means of 3 separate experiments ± standard deviation (*n* = 8, each).

Figure 2 gives the percentages of liposomes, which had been taken up, calculated from applied and cell lysate concentrations. Highest uptake efficiency occurred between 75 and 175 µM. At an applied concentration of 100 µM, the liposomes in the cell lysate corresponded to 12.8% ± 2.3%, at 1 mM only to 4.3% ± 0.5%.

Higher concentrations of cBSA-liposomes led to higher final concentrations in the cell lysate, which was to be expected. However, the highest uptake efficiency of approximately 13% was seen at 100 µM. Possibly, higher concentrations cause the membrane to become saturated, leading to a partially reduced uptake because of the limited endocytotic capacity of the cells. Not enough liposome cell contact could result in a lower uptake at lower applied concentrations, as more liposomes stay dispersed.

**Figure 3:**
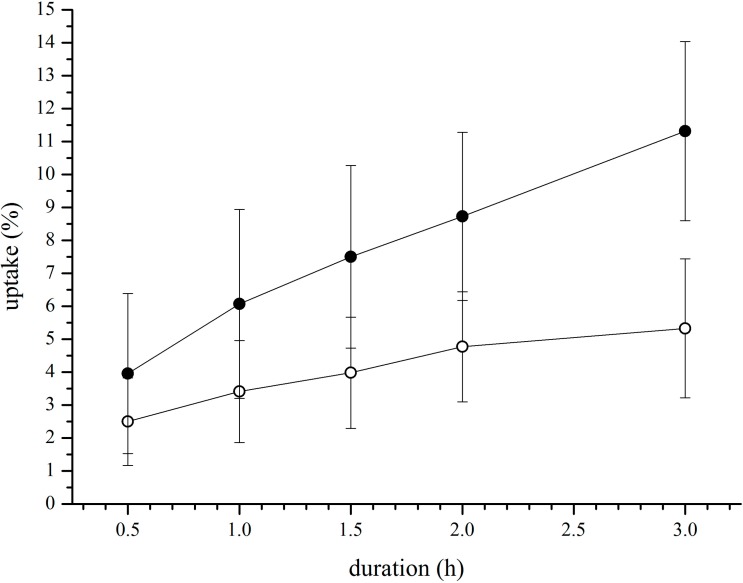
Percentage of liposomes taken up after incubation with 100 µM (solid symbols) and 250 µM (open symbols) of cBSA-liposomes for different timeframes. Values are the means of 3 separate experiments ± standard deviation (*n* = 8, each).

In order to get a better understanding of the uptake kinetics time resolved, uptake experiments were performed at 100 and 250 µM cBSA-liposomes over a time interval of 3 h. Percentages of intracellular liposomes are shown in Figure 3, where 11.3% ± 2.8% were reached for 100 µM and 5.3 ± 2.2 for 250 µM. In Table 2, the values for uptake rates determined by linear regression are given.

**Table 2:** Percental and molar uptake rates for 100 und 250 µM cationized bovine serum albumin (cBSA)-liposomes. Values and errors are determined by linear regression.

Concentration	Percental Rate (%/h)	Molar Rate (µM/h)
100 µM	2.9 ± 0.2	2.9 ± 0.2
250 µM	1.1 ± 0.2	2.8 ± 0.5

While the uptake of liposomes at 100 µM again shows a higher efficiency, with a percental rate almost triple that of 250 µM, the molar uptake rate of both is approximately the same. Over the whole of three hours, the uptake seems to occur in a linear fashion. An asymptotic behaviour that would allow for the assumption of a possible saturation effect can not be observed. Higher concentrations might show this within 3 h but were not tested. Experiments longer than three hours are not possible due to the sensitivity of the cells.

To elucidate the manner of endocytosis uptake, experiments with known endocytotic inhibitors were performed, namely: dansylcadaverine (100 µM), filipin (3 µg/mL), nocodazol (4 µM), phenylarsinoxide (20 µM) and chlorpromazine (14 µM). The results repeated our previous findings [6], which showed inhibition through 3 µg/mL filipin (45% ± 5% inhibition), 4 µM nocodazol (50% ± 18% inhibition) and 20 µM phenylarsinoxide (75% ± 7% inhibition), being indicative for a caveolae associated mechanism of endocytosis [30]. Aside from these inhibitors, the effects of an excess of free BSA and free cBSA on liposomal uptake were investigated. In Table 3, the inhibitory effects of these experiments are listed. The excess is given with regard to liposomal protein if all linkers were occupied.

**Table 3:** Inhibitory effects of free bovine serum albumin (BSA) and cBSA. Values are derived from mean of inhibited and uninhibited samples of 3 separate experiments ± standard deviations (*n* = 8, each). Statistical analysis was performed via *t* test (***, *p* < 0.001).

BSA 10fold (%)	BSA 100fold (%)	cBSA 10fold (%)	cBSA 100fold (%)
15.5 ± 2.0	49.5 ± 10.7	22.9 ± 6.9 ***	67.7 ± 6.2 ***

At a 10fold excess, cBSA shows a slightly higher inhibitory effect than BSA, which became even more evident at a 100fold excess. In both cases, the increase in inhibition is statistically significant. An increasing membrane saturation caused by these proteins seems to be the probable cause for this inhibition. Being positively charged, cBSA should have a higher membrane affinity, which seems to be corroborated through higher inhibitory effects.

As a control, the uptake of liposomes without any proteins and liposomes conjugated with native BSA was tested. Both liposomes with PEGylated lipids and without showed only uptakes above an applied concentration of 1 mM, with BSA-liposomes already at 250 µM. Lower concentrations showed no measurable uptake and higher ones were not tested. The resulting percental uptakes and their comparison with the according values for cBSA-liposomes are shown in Table 4.

**Table 4:** Improvement through cBSA attachment to liposomes. Values are the means of 2 (blank and PEG-liposomes) and 3 (BSA- and cBSA-liposomes) separate experiments ± standard deviation (*n* = 8, each). Errors of improvement factors were determined using error propagation. Statistical analysis was performed via *t* test (***, *p* < 0.001).

Liposome Type	Uptake (%)	According cBSA-Liposome Uptake (%)	cBSA Improvement Factor
Blank liposomes (1 mM)	1.3 ± 0.5 ***	4.3 ± 0.5	3.3 ± 1.7
PEG-liposomes (1 mM)	0.9 ± 0.4 ***	4.3 ± 0.5	4.8 ± 2.7
BSA-liposomes (250 µM)	0.030 ± 0.006 ***	9.7 ± 2.1	323.3 ± 134.7

Compared to unconjugated liposomes, cBSA-liposomes show uptake at far lower concentrations. Even at the lowest concentration, where uptake for blank and PEG-liposomes could be seen, cBSA-liposomes were still 3–4 times more effective. The uptakes of BSA-liposomes was detectable at lower concentrations than that of unconjugated liposomes, but they were still not as low as that of cBSA-liposomes. The latter, however, showed a tremendously better uptake, thus substantiating the improvement achieved by the cationization of BSA.

**Figure 4:**
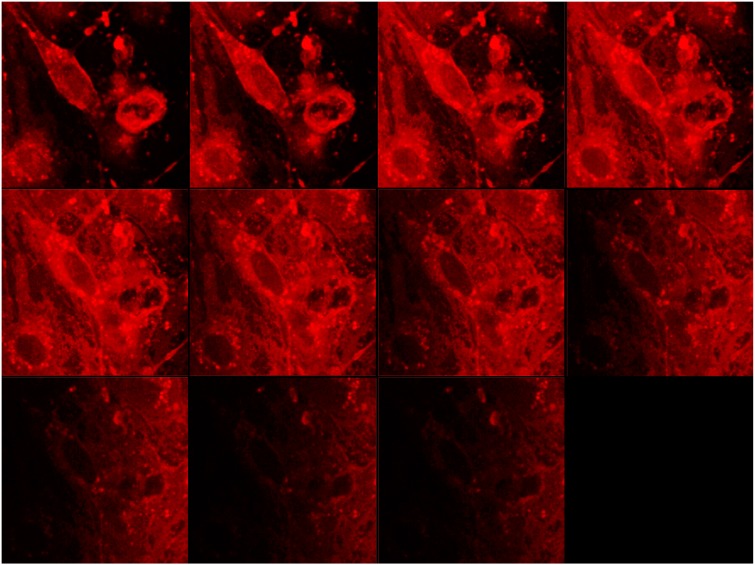
Fluorescence images of live porcine brain capillary endothelial cells (PBCEC) in z-stack incubated with cBSA-liposomes at 100 µM. Steps between images were 20 µM.

In order to confirm the actual uptake of cBSA-liposomes into PBCEC, confocal laser scanning microscopy images were taken of live cells incubated with 100 µM. To ensure that liposomes have indeed been taken up and are not only bound to the surface of the liposomes, z-stack image series were performed, which are shown in Figure 4. They demonstrate that fluorescence accumulated inside the cells in a punctuated pattern with cell nuclei clearly distinguishable, suggesting vesicular compartmentation of the endocytosed material.

### 3.3. In Vivo Uptake of cBSA-Liposomes

**Figure 5:**
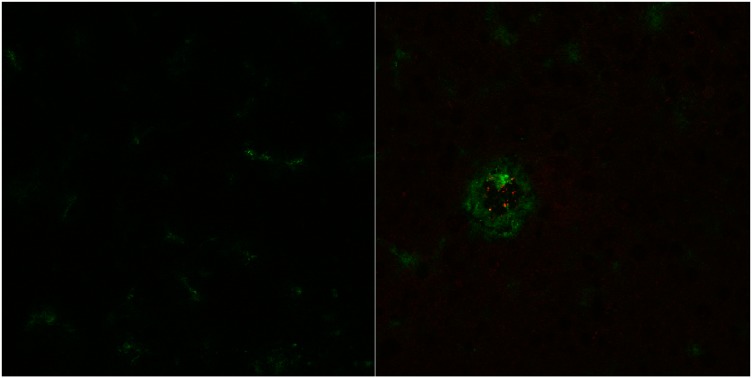
Exemplary image of rat brain cryosection after 1 h of cBSA-liposome incubation (**right**) with control (**left**). Capillaries have been stained with fluorescein labelled cBSA (FITC-BSA) (green). Liposomal rhodamine B (red).

Cryosections of rat brains one hour after application of cBSA-liposomes show a distinct pattern of red fluorescence derived form the rhodamine B incorporated into the liposomes when compared to cryosections of rats that did not receive them. In Figure 5, an exemplary image is shown in comparison to its control. The applied liposomes were of a diameter of 122.0 ± 0.8 nm with a polydispersity index (PDI) of 0.31 ± 0.04. The zeta potential amounted to 6.6 ± 0.4 mV and the dose to 21.9 ± 3.2 mg lipid per kg body weight, which corresponds to a plasma concentration of 0.35 ± 0.06 mg/mL of lipid, calculated under the assumption the blood amount of a rat is 6% of its body mass.

The spotty fluorescence seen in Figure 5 allows the conclusion that the liposomes are still intact. The liposomal fluorescence is mainly associated with the lumen of the capillary. In the interstitial space, none can be observed, suggesting that no significant permeation into brain tissue has occurred yet.

**Figure 6:**
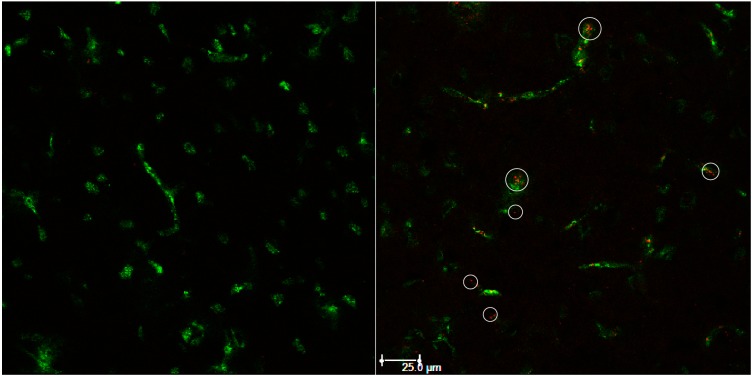
Exemplary image of rat brain cryosection after 3 h of cBSA-liposome incubation (**right**) with control (**left**). Capillaries have been stained with FITC-BSA (green). Liposomal rhodamine B (red).

To further characterize the process of liposome uptake, cryosections after different times of incubation were investigated. As can be seen in Figure 6, three hours after liposome application, most detectable liposomes appear to be in close proximity to the brain capillary walls and beyond. The characteristics of the applied liposomes were a diameter of 132.8 ± 1.3 nm with PDI 0.19 ± 0.02, a zeta potential of 3.7 ± 3.3 mV and an applied dose of 14.77 ± 0.08 mg/kg (0.233 ± 0.002 mg/mL). A second experiment carried out with liposomes of 182.4 ± 4.5 nm diameter with PDI 0.156 ± 0.009, zeta potential 7.7 ± 0.5 mV and applied to a dose of 11.6 ± 2.0 mg/kg (0.183 ± 0.04 mg/mL), did not show liposomal fluorescence (image not shown).

**Figure 7:**
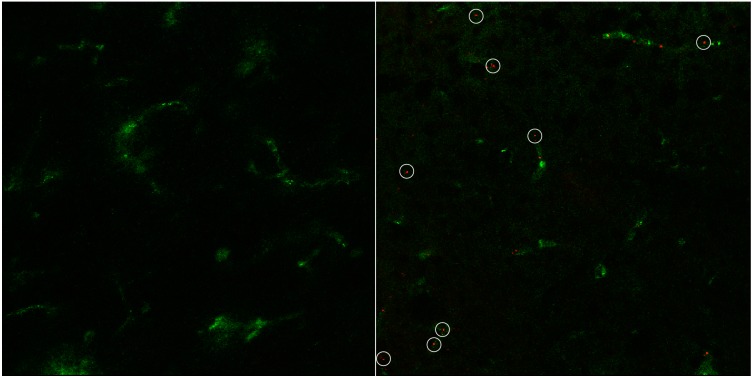
Exemplary image of rat brain cryosection after 6 h of cBSA-liposome incubation (**right**) with control (**left**). Capillaries have been stained with FITC-BSA (green). Liposomal rhodamine B (red).

Figure 7 gives the cryosections for an incubation of six hours (122.0 ± 0.8 nm, PDI 0.31 ± 0.04, 6.6 ± 0.4 mV, 22.6 ± 1.7 mg/kg, 0.36 ± 0.03 mg/mL). There are still several spots of liposomal fluorescence detectable. A second experiment performed (182.4 ± 4.5 nm, PDI 0.156 ± 0.009, 7.8 ± 0.5 mV) with a dose of 11.7 ± 1.9 mg/kg (0.19 ± 0.03 mg/mL) again did not lead to any detectable liposomal fluorescence after six hours.

**Figure 8:**
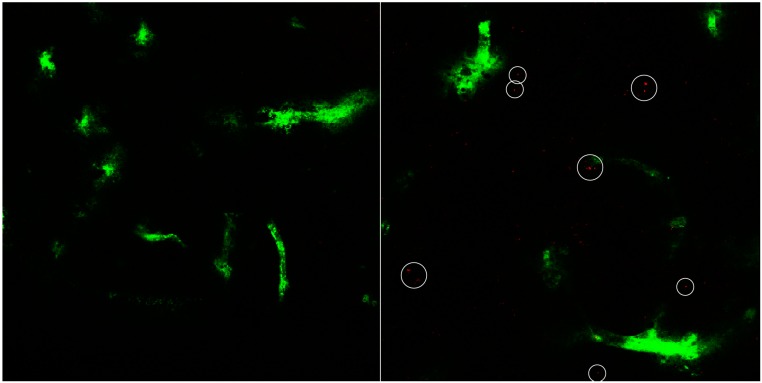
Exemplary image of rat brain cryosection after 24 h of cBSA-liposome incubation (**right**) with control (**left**). Capillaries have been stained with FITC-BSA (green). Liposomal rhodamine B (red).

Finally, as can be seen in Figure 8, 24 h after application, liposomal fluorescence is still visible in an amount comparable to six hours. Where there is red fluorescence, however, it is mostly not capillary associated any more, which would mean liposomes have moved into the brain tissue itself. The liposomes applied were 140.3 ± 1.1 nm in diameter with a PDI of 0.28 ± 0.02 and a zeta potential of 8.4 ± 0.7 mV. The applied dose amounted to 24.1 ± 0.4 mg/kg (0.381 ± 0.006 mg/mL).

The amount of fluorescence visible in the brain cryosections peaks at 3 h incubation time and declines towards 6 h and then stays approximately the same till 24 h. At one hour, liposomal fluorescence can not be detected outside the capillary lumen. After 24 h, the remaining fluorescence is mostly in the space between capillaries, suggesting that uptake of cBSA-liposomes occurs mainly between 1 and 3 h. Afterwards, the uptake apparently slows down and simultaneously occurring degradation of liposomes leads to decreasing fluorescence over 24 h.

**Figure 9:**
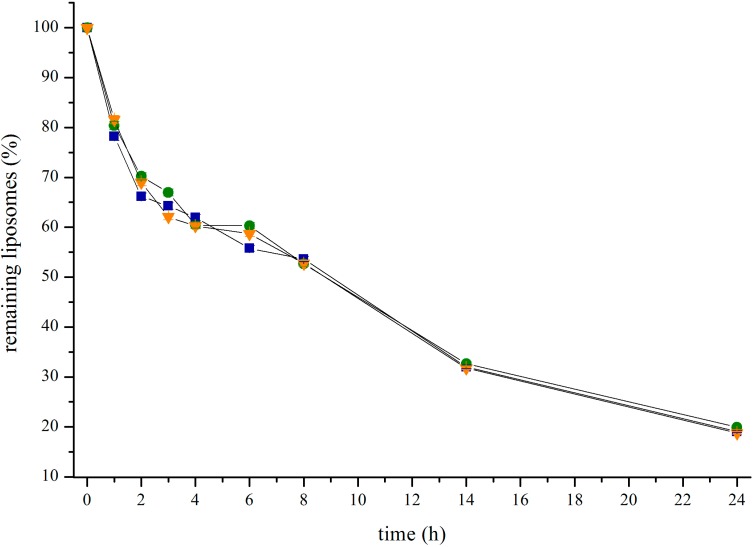
Remaining liposomes in the bloodstream of three rats over a time course of 24 h.

In order to learn more on the plasma lifetime of cBSA-liposomes, blood samples were taken during a 24 h experiment. In Figure 9, the remaining percentage of liposomes in the bloodstream is given. After a period of three hours, approximately 60% to 70% of the liposomes were still circulating. At the end of the experiment at 24 h, there were still around 20% left in the bloodstream. Exponential fitting yields a half life of cBSA-liposomes of 8.6 ± 0.2 h.

The clearance of cBSA-liposomes over 24 h is low and seems to be slow enough to permit the uptake into brain tissue. It stands to reason that, aside from the brain, other organs are involved in liposome uptake as well.

## 4. Conclusions

The present data are in good accordance with our previous data with ApoE and anti-transferrin-receptor antibody modified liposomes [17,31], which demonstrated uptake and permeation of liposomal delivery systems across the BBB *in vitro* and *ex vivo*. The images derived from the *in vitro* and *in vivo* experiments from this study clearly indicate that cBSA-liposomes are able to enter PBCEC and to move on into brain tissue. Actual penetration into brain tissue based on images alone, however, is subjective and difficult to discern. Therefore, experiments are ongoing in order to quantify the actual uptake of the applied dose of liposomes into the brain in conjuncture with a capillary depletion technique to avoid detection of liposomes stuck in the capillary wall. Aside from this, cBSA-liposomes appear to be a promising tool in drug delivery to the central nervous system.
